# All-Fiber Flexible Electrochemical Sensor for Wearable Glucose Monitoring

**DOI:** 10.3390/s24144580

**Published:** 2024-07-15

**Authors:** Zeyi Tang, Jinming Jian, Mingxin Guo, Shangjian Liu, Shourui Ji, Yilong Li, Houfang Liu, Tianqi Shao, Jian Gao, Yi Yang, Tianling Ren

**Affiliations:** 1 School of Integrated Circuit, Tsinghua University, Beijing 100084, China; 2 Beijing National Research Center for Information Science and Technology (BNRist), Tsinghua University, Beijing 100084, China; 3 BOE Health Technology Co., Ltd., Beijing 100016, China

**Keywords:** all-fiber, electrospinning, electrochemical sensor, glucose continuous monitoring

## Abstract

Wearable sensors, specifically microneedle sensors based on electrochemical methods, have expanded extensively with recent technological advances. Today’s wearable electrochemical sensors present specific challenges: they show significant modulus disparities with skin tissue, implying possible discomfort in vivo, especially over extended wear periods or on sensitive skin areas. The sensors, primarily based on polyethylene terephthalate (PET) or polyimide (PI) substrates, might also cause pressure or unease during insertion due to the skin’s irregular deformation. To address these constraints, we developed an innovative, wearable, all-fiber-structured electrochemical sensor. Our composite sensor incorporates polyurethane (PU) fibers prepared via electrospinning as electrode substrates to achieve excellent adaptability. Electrospun PU nanofiber films with gold layers shaped via thermal evaporation are used as base electrodes with exemplary conductivity and electrochemical catalytic attributes. To achieve glucose monitoring, gold nanofibers functionalized by gold nanoflakes (AuNFs) and glucose oxidase (GOx) serve as the working electrode, while Pt nanofibers and Ag/AgCl nanofibers serve as the counter and reference electrode. The acrylamide-sodium alginate double-network hydrogel synthesized on electrospun PU fibers serves as the adhesive and substance-transferring layer between the electrodes. The all-fiber electrochemical sensor is assembled layer-by-layer to form a robust structure. Given the stretchability of PU nanofibers coupled with a high specific surface area, the manufactured porous microneedle glucose sensor exhibits enhanced stretchability, superior sensitivity at 31.94 μA (lg(mM))^−1^ cm^−2^, a broad detection range (1–30 mM), and a significantly low detection limit (1 mM, S/N = 3), as well as satisfactory biocompatibility. Therefore, the novel electrochemical microneedle design is well-suited for wearable or even implantable continuous monitoring applications, thereby showing promising significant potential within the global arena of wearable medical technology.

## 1. Introduction

Accompanied by the development of wearable sensors and flexible material preparation technology, wearable monitoring methods based on electrochemical and optical sensors have substantially advanced [1,2,3,4]. However, due to the complexity of the SERS-based optical system, it is not easy to achieve portable detection, and commercial optical wearable devices cannot provide high precision compared with electrochemical sensors. Wearable electrochemical glucose detection has been extensively developed for use within the scope of laboratories and commercial products [5,6,7,8,9]. Gao Wei et al. have prepared a flexible skin patch sensing system by exploiting micro-nanofabrication techniques [10]. Joseph Wang and his team have leveraged 3D printing technology to create a wearable microneedle array sensor capable of concurrently monitoring multiple physiological indicators [11]. Additionally, corporations such as Abbott and Dexcom have introduced continuous glucose monitoring (CGM) products that can continuously monitor glucose levels in the interstitial fluid (ISF), thereby providing a representation of blood glucose levels [12].

However, the wearable system designs mentioned above still have some issues that need to be resolved to improve the systems’ monitoring capabilities in non-laboratory environments. For example, patch electrodes based on micro/nano fabrication methods have high requirements for the preparation method, and implantable commercial sensors based on hard microneedles can cause skin reactions after implantation due to the rigid PET substrate used [13]. To resolve these issues, researchers need a simple method of preparing electrodes that can conform to skin tissue deformation making them flexible for electrochemical use. Electrospinning technology is a simple method of preparing high-elasticity nanofiber films with high specific surface areas to obtain wearable and implantable electrochemical electrodes [14].

In this paper, we have designed an all-fiber wearable glucose sensor prepared by an electrospinning process. Using thermal evaporation, we prepared a dense gold film on PU mesh constructed by electrospinning, achieving good conductivity. Additionally, AuNFs were deposited on the working electrode to enhance the electrochemical activity of the electrode. The AuNFs, in conjunction with the PU electrospun fiber’s porous mesh structure, provide abundant active sites for electrode reactions. The optimal concentration of AuNFs was determined to optimize the effective active area of the electrode. Moreover, modifying the electrode with Prussian Blue (PB) as a mediator for electron transfer in the enzyme reaction significantly reduced the potential applied on the electrode, enhancing electrode stability and reducing interference [15]. The electrodes’ surface morphology and electrochemical performance were characterized using scanning electron microscope (SEM), cyclic voltammetry, and electrochemical impedance spectroscopy (EIS). In the layer-by-layer assembly process of the electrode, a double network hydrogel based on acrylamide and sodium alginate, reinforced by the electrospun mesh, was introduced to serve as an adhesive and a substance-transferring layer between the electrodes. Furthermore, due to the flexibility of the electrospun film, the wearable sensor system can maintain stable conductivity while conforming to skin deformation. The biocompatibility of the electrode was also evaluated, and a high degree of biocompatibility was observed, further proving its potential for practical applications. The sensor proposed in this study can advance research in wearable glucose detection and promote the development of wearable sensors.

## 2. Materials and Methods

### 2.1. Materials

The PU solution was prepared by mixing PU particles (Tecoflex SG-85A, Shanghai, China) and N, N-dimethylformamide (from Aladdin, Shanghai, China), and Tetrahydrofuran (from Adamas, Shanghai, China) at a weight ratio of 15:42.5:42.5 before magnetic stirring for 3 h at 55 °C to form a homogeneous transparent bubble-free solution. The PU is chemically pure, and tetrahydrofuran and dimethyl formamide are anhydrous. The acrylamide and sodium alginate, gold (III) chloride trihydrate and tetramethyl-ethylenediamine iron (III) chloride, potassium chloride, potassium ferricyanide (III), hydrochloric acid, GOx, Au nanoparticle (AuNPs), chitosan, glutaric dialdehyde, Nafion, uric acid (UA), ascorbic acid (AA) were purchased from Sigma-Aldrich, Shanghai, China. The N, N-methylene bisacrylamide, and ammonium persulfate were purchased from Adamas. The calcium carbonate nanopowder was purchased from Zkynxc (Beijing, China). Dulbecco’s Modified Eagle Medium, 0.25% trpsin-EDTA, fetal bovine serum, human skin fibroblasts HSF T25, and phosphate-buffered saline (PBS) solution were purchased from Gibco, Shanghai, China. Penicillin-Streptomycin (100×) was purchased from TransGen, Beijing, China. A Live/Dead Cell Double Staining Kit and NucBlue Live Ready Probes were purchased from Invitrogen, Shanghai, China.

### 2.2. Fabrication of PU Nanomesh and Au Nanomesh and Pt/Au Nanomesh and Ag/AgCl/Au Nanomesh

The electrospinning PU solution was mentioned above. The prepared solution was added into a 10 mL plastic syringe using a 26G needle. The electrospun PU mesh was collected using a metallic roller with aluminum foil as the collector. During the electrospinning procedure, the applied voltage, electrospinning time, distance between needle and collector, spinning rate of collector, and pump rate were 15 kV, 150 min, 20 cm, 100 rpm, and 0.2 mm min^−1^, respectively. Subsequently, we removed the electrospun film from the foil using a PET frame and placed the frame on a flat silicon wafer using double-sided adhesive. First, to obtain Au/PU nanomesh, a layer of Au film with a thickness of 100 nm was thermally evaporated onto one side of the PU mesh servers as the working electrode. To increase the stability of our electrode, a 100 nm Pt layer and a Ag layer were thermally evaporated on the Au/PU nanomesh. The Pt/PU nanomesh was used as the counter electrode. The Ag/AgCl/Au/PU electrode was obtained by immersing the Ag/Au/PU nanomesh into the 0.1 M FeCl_3_ for 1 min to prepare the reference electrode.

### 2.3. Optimization and Functionalization of Electrode

After the thermal evaporation of Au, this original Au working electrode was further optimized by electrodeposited AuNFs. AuNFs were electrodeposited in situ by cyclic voltammetry from −1 V to 0 V (versus Ag/AgCl) for 20 cycles at the scan rate of 0.02 V s^−1^ in solution with HAuCl_4_ and phosphate buffer on the working electrode with a Pt electrode and Ag/AgCl electrode as counter and reference electrode, respectively. The concentration of HAuCl_4_ was investigated to determine the optimal electrochemical performance. The AuNFs/Au/PU electrode was further functionalized to fulfill the need to monitor glucose. First, a layer of PB was electrodeposited on the working electrode by cyclic voltammetry from 0.5 V to 1.1 V for 20 cycles at 0.02 V s^−1^ in the solution with 2.5 mM FeCl_3_, 100 mM KCl, 2.5 mM K_3_Fe(CN)_6_, and 100 mM HCl. To obtain the glucose sensor, 10 μL of the GOx/chitosan/AuNPs mixture was drop-casted onto the PB/AuNFs/Au/PU working area. The mixture contained 30 mg mL^−1^ GOx, 1 wt% chitosan, and 5 mg mL^−1^ AuNPs. The modified sensor was allowed to dry at 4 °C for about 24 h. Then, 10 μL of 0.5 wt% Nafion solution was drop-casted on the working electrode. After drying in an ambient atmosphere, the electrode was treated with 2 wt% glutaraldehyde for 30 s. The obtained electrode was thoroughly rinsed with 10× PBS and stored at 4 °C when not in use.

### 2.4. Preparation of Hydrogel/PU Nanomesh

The hydrogel precursor was prepared by dissolving 4 wt% acrylamide, 0.52 wt% sodium alginate, 0.0025 wt% N, N-methylene bisacrylamide, 0.069 wt% calcium carbonate nanopowder, 0.039 wt% ammonium persulfate, and 0.26 wt% tetramethyl-ethylenediamine in deionized water. Then, PU mesh was immersed into the fully degassed precursor. Thermal curing was conducted at 60 °C for 2.5 h to obtain the hydrogel/PU nanomesh.

### 2.5. Cell Culture Procedure

HSF-T25 cells were grown in Dulbecco’s Modified Eagle Medium (DMEM) with 10% FBS, and 1× penicillin/streptomycin in a sterile CO_2_ incubator at 37 °C and 5 % CO_2_. When the density of the cells reached 60–70 %, subculture treatment was considered. Cells were transferred into a 12-well plate for 24 h before the experiment. The density was 8 × 10^4^ cells per well.

### 2.6. Characterization and Testing

Scanning electron microscope (SEM) and energy-dispersive X-ray spectroscopy (EDS) measurements were performed using JCM-7000 (JEOL, Tokyo, Japan) and GeminiSEM 300 (ZEISS, Oberkochen, Germany). Laser cutting was completed using the 50 W CO_2_ laser cutter Universal VLS2.30DT (Universal Laser System, Arlington Heights, IL, USA). The electrochemical tests, including cyclic voltammetry, chronoamperometry, and EIS, were implemented using PGSTAT302N (Autolab, Arbon, Switzerland). EIS was conducted at a frequency range of 0.01 Hz–100 kHz by applying a sinusoidal signal with an amplitude of 5 mV in 0.1 M KCl containing 5 mM [Fe(CN)_6_]^3−/4−^. The measured EIS spectra were fitted to an equivalent circuit model (Figure S2, insert). The test configuration was in a 3-probe system with Pt as the counter electrode and Ag/AgCl as the reference electrode. Glucose solutions at varied concentrations were obtained based on 10× PBS (pH 7.2~7.4). Chronoamperometry was conducted at 0.05 V. The cells were observed using a confocal microplate imaging detection system (BioTek Cytation C10, Agilent, Beijing, China). The sensors were stored at 4 °C in PBS when not in use.

## 3. Result and Discussion

### Preparation of Standalone Electrodes

**Figure 1:**
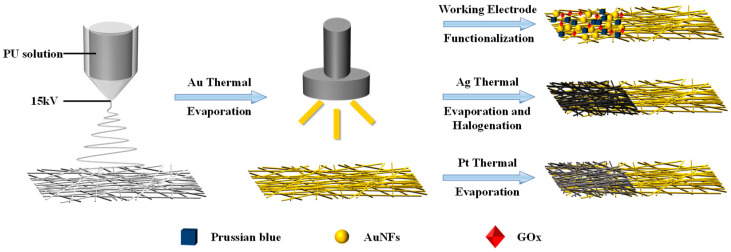
Diagram of the mesh fabrication procedure.

Figure 1 shows the fabrication method of standalone electrodes based on electrospinning fiber, with the specific fabrication parameters detailed in Materials and Methods.

**Figure 2:**
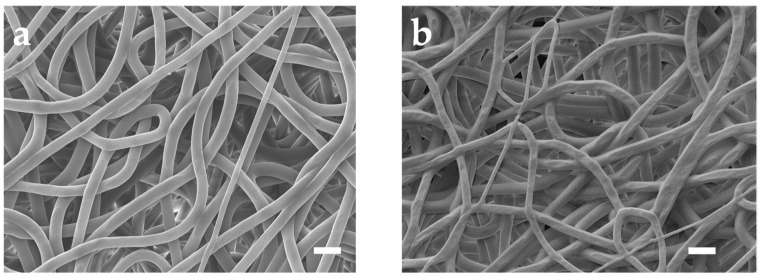
SEM images of the (**a**) untreated PU mesh and (**b**) Au-coated PU mesh. Scale bars, 10 μm.

Figure 2a depicts the SEM photo of untreated PU mesh. Due to the electric field formed by the high voltage applied between the needle containing the PU solution and the collector during the electrospinning process, the electrostatic repulsion force overrides the surface tension. Accompanied by the evaporation of the solvent, a mesh structure constructed with randomly oriented PU nanofibers forms on the collector [16]. Then, we removed the mesh from the aluminum foil using a PET frame with a thickness of 125 μm. The obtained PU mesh was placed flat on a silicon wafer with the assistance of PI tape. Then, the wafer with PU mesh was fixed in the evaporation chamber. After that, a 100-nm-thick Au layer was thermally evaporated on the surfaces of the wafer as well as PU mesh at a speed of 1 Å/s. Removing the PI tape allows the silicon wafer and PU mesh to be easily separated without destroying Au-coated fibers. The stacked nanofibers covering Au can form a well-connected conductive network. The different fiber surfaces can be observed in the SEM image of the Au mesh (Figure 2b). In addition, a layer of Pt and Ag was thermally evaporated onto the Au layer to enhance the integrated electrodes’ electrochemical stability. The Ag/Au/PU electrode was then treated with FeCl_3_ solution to obtain Ag/AgCl as the reference electrode.

**Figure 3:**
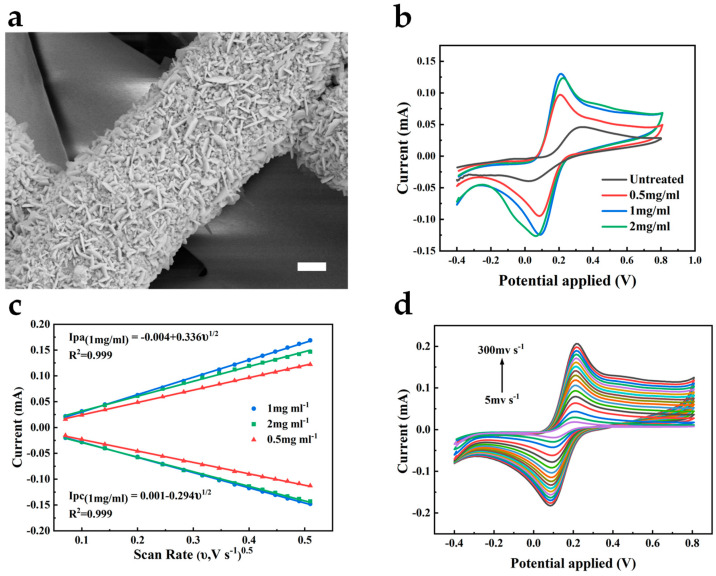
(**a**) SEM image of the AuNFs-deposited Au/PU mesh. Scale bar, 1 μm. (**b**) CV curves of different treated electrodes in 0.1 M KCl containing 5 mM [Fe(CN)_6_]^3−/4−^. Scan rate 50 mV s^−1^. (**c**) The calibration plot of the peak current versus the square root of the scan rate of different treated electrodes. (**d**) CV curves of 1 mg mL^−1^ treated electrode at different scan rates in 0.1 M KCl containing 5 mM [Fe(CN)_6_]^3−/4−^.

Here, to enhance the electrochemical catalytic ability of the bare gold electrode, we chose to modify the electrode with Au nanomaterials. Due to their large specific surface area, high conductivity, and biocompatibility, their attachment to the electrode benefits the acceleration of the electron transfer rate in the electrode reaction, thus amplifying the response current and related sensitivity [17]. As shown in Figure 3a, Au nanoflakes are uniformly deposited on the Au/PU nanofibers, which further increase the specific surface area of the electrode. To validate the role of AuNFs, we performed cyclic voltammetry measurements on the untreated Au electrode and the electrode after the deposition of AuNFs using the [Fe(CN)_6_]^3−/4−^ redox probe, as shown in Figure 3b. We can observe the quasi-reversible behavior of the redox probe on both electrodes. Compared to the bare Au electrode, the AuNFs-treated electrode shows a larger oxidation–reduction current. It reflects the enhanced electrocatalytic ability of the electrode by the electrodeposited AuNFs, thereby improving the electrochemical active surface area of the electrode. The fiber width of the electrode increased with the HAuCl_4_ concentration and reached its most prominent value at a concentration of 2 mg mL^−1^ (Figure S1).

According to the electrochemical surface area, the HAuCl_4_ concentration was studied to optimize the electrodeposited AuNFs. The AuNFs-treated electrode showed the most significant response current and the smallest peak-to-peak separation when the HAuCl_4_ concentration was 1 mg mL^−1^ (Figure 3b). We also measured the cyclic voltammograms of the AuNFs/Au/PU electrodes deposited at different HAuCl_4_ concentrations under different scan rates. Compared with other concentrations, the peak current of the electrode pretreated at 1 mg mL^−1^ showed a higher slope to the square root of the scan rate, as shown in Figure 3c. According to the Randles–Sevcik equation, the electrode treated at a concentration of 1 mg mL^−1^ can exhibit a larger electrochemical active surface area, demonstrating that the oxidation-reduction reaction on the electrode is a diffusion-controlled process. Simultaneously, this electrode can still maintain the shape of the cyclic voltammetry curves at a high scanning speed of 300 mV s^−1^, reflecting the high efficiency of electron transfer on the electrode surface and the excellent conductivity of the electrode, as shown in Figure 3d. According to the EIS spectra of electrodes deposited by different concentrations of HAuCl_4_ (Figure S2), the electrode treated with 1 mg mL^−1^ of HAuCl_4_ showed the lowest charge transfer resistance (R_ct_ = 77 Ω), whereas the R_ct_ of the 2 mg mL^−1^ treated electrode was 90 Ω and that of the 0.5 mg mL^−1^ treated electrode was 176 Ω. The result implies that electrodes treated with 1 mg mL^−1^ HAuCl_4_ are more feasible for electron transfer.

**Figure 4:**
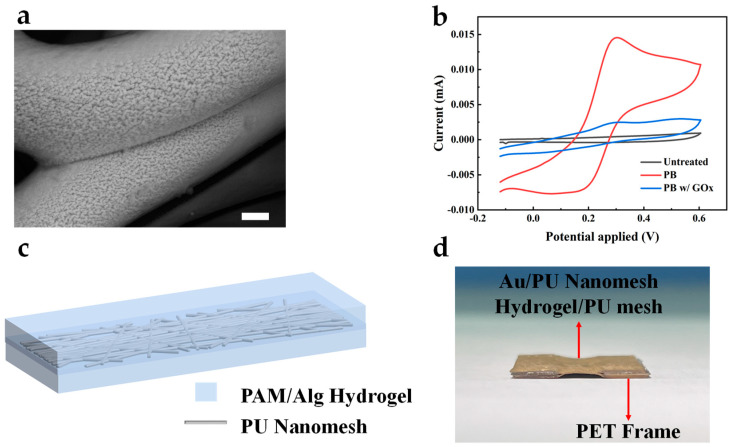
(**a**) SEM image of the PB-deposited AuNFs/Au/PU mesh. Scale bar, 1 μm. (**b**) CV curves of GOx/PB/AuNFs/Au/PU, PB/AuNFs/Au/PU, and AuNFs/Au/PU electrode in 10× PBS. (**c**) Schematic of electrospun mesh-reinforced hydrogel. (**d**) The optical images of the five-layer structure after 2 weeks immersed in deionized water.

The enzymatic oxidation of glucose mediated by GOx generates hydrogen peroxide (H_2_O_2_) as a byproduct. PB is renowned for its electrocatalytic behavior towards H_2_O_2_, making it suitable for the AuNFs/Au/PU electrode mentioned above. Figure 4a shows the surface morphology of PB deposited on the electrode via cyclic voltammetry. Compared to the uncoated bare electrode, the PB-modified electrode showed a wrinkled surface, indicating the successful electrodeposition of PB. The electrochemical characterization of the GOx/PB/AuNFs/Au/PU electrode, PB/AuNFs/Au/PU electrode, and bare Au electrode in PBS solution was performed using cyclic voltammetry, as shown in Figure 4b. Due to the lack of an electrochemical redox probe on the bare gold electrode, no evident redox current peak is observed in its cyclic voltammetry curve. However, upon the electrode’s coating with PB, the reversible electrochemical behavior of PB on the electrode is observed in the PBS solution, indicating the successful deposition of PB on the electrode. To assemble the standalone electrospun electrodes into an all-fiber three-electrode system, we introduced a double-network hydrogel based on polyacrylamide-sodium alginate as an adhesive and substance-transferring layer for the independent electrodes. An electrospun mesh was incorporated into the hydrogel system to enhance its mechanical strength. Figure 4c shows the schematic of the electrospun mesh-reinforced hydrogel, and the preparation method is presented in Materials and Methods. To evaluate the adhesion performance of the hydrogel, Au/PU electrodes carried by laser-cut PET were assembled into a five-layer structure (A/H/A/H/A) using the prepared hydrogel and placed in deionized water. After 2 weeks, the structure maintained stable adhesion and good conductivity, as shown in Figure 4d. This demonstrates that the hydrogel could be applied to long-term monitoring while retaining good adhesion.

**Figure 5:**
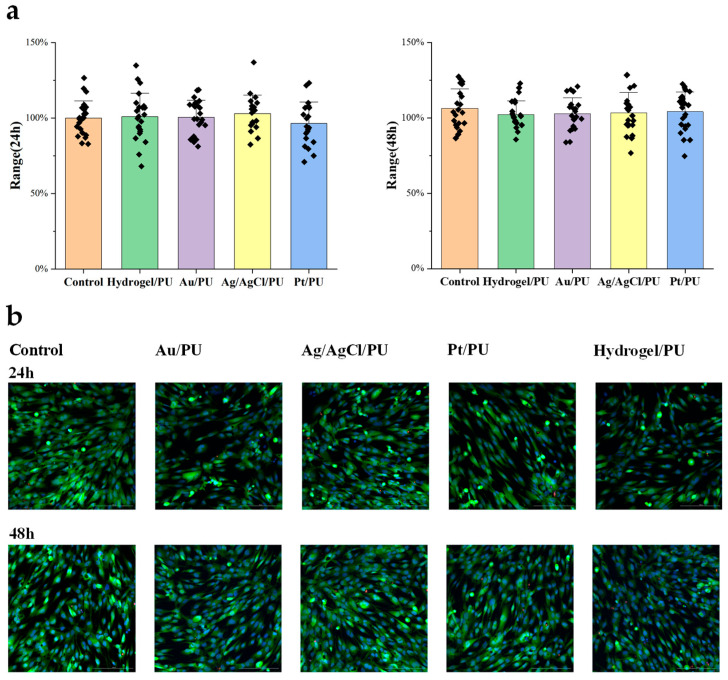
(**a**) Cell numbers after 24 h (**left**) and 48 h (**right**) treatment. (**b**) Representative fluorescence images of different treated cells.

To evaluate the biocompatibility of the all-fiber sensor, we designed an experiment to study the cytotoxicity of the meshes assembled into the sensor. The Materials and Methods section shows the specific cell culture and passage steps. First, we cultured human skin fibroblasts in a 12-well plate until they covered the bottom of the dish. Then, we added approximately 5 mm^2^ of the mesh to be tested to the culture dish. After 24 h and 48 h, we took images and counted the cells. After co-culturing with the mesh for 48 h, we did not observe significant differences in cell morphology and cell proliferation activity compared to the control group. Therefore, the cytotoxicity results indicate that the resultant meshes have good biocompatibility and meet the requirements for further implantable monitoring (Figure 5).

**Figure 6:**
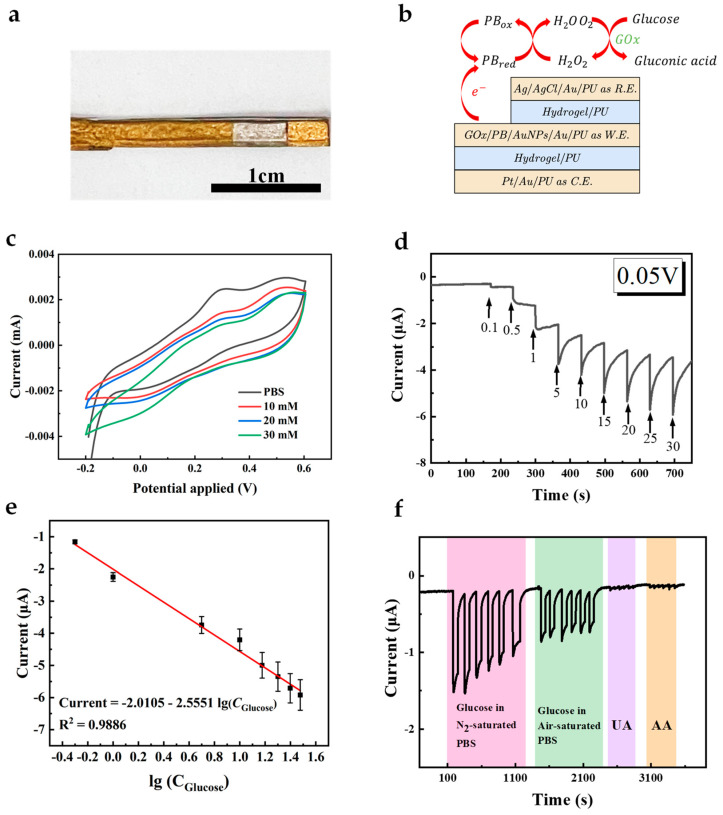
(**a**) The optical images of the composite electrode. Scale bar, 1 cm. (**b**) The diagram of the sensor. (**c**) CV curve of the GOx/PB/AuNFs/Au/PU electrode in PBS with and without 10 mM glucose at a scan rate of 50 mV s^−1^. (**d**) Amperometric response of the GOx/PB/AuNFs/Au/PU electrode in glucose solutions with different concentrations ranged from 0 mM to 30 mM. (**e**) The calibration plot of the response current on GOx/PB/AuNFs/PU electrode (n = 3). (**f**) Amperometric response of the GOx/PB/AuNFs/PU towards 5 mM glucose in air/N_2_-saturated PBS and 5 mM UA, 0.1 mM AA in N_2_-saturated PBS.

The successful modification of AuNFs, PB, and GOx on the working electrode was demonstrated. To enhance the electron exchange rate between the active sites of GOx and the working electrode, GOx was mixed with water-dispersed AuNPs to modify the working electrode. Figure 6a,b show the conceptual schematic and pictures of the all-fiber electrode assembled using the hydrogel-PU mesh, with the independent electrodes acting as working electrode, counter electrode, and reference electrode, forming a whole sensor system. The sensing principle based on PB and GOx is illustrated in Equations (1)–(5). In the absence of oxygen (Equations (1), (2) and (5)), i.e., in an N_2_-saturated environment, glucose is first catalyzed by GOx to gluconic acid, with the flavin adenine dinucleotide (FAD) cofactor in GOx being converted to the reduced cofactor flavin adenine dinucleotide (FADH_2_). PB, acting as the mediator, reacts with FADH_2_ to form PB in its reduced form (PB_red_). Subsequently, PB_red_ can be oxidized at the electrode surface, allowing continuous cycling of the mediator and enabling the glucose oxidation process to proceed. In the presence of oxygen (Equations (1) and (3)–(5)), the reaction in Equation (1) occurs similarly, followed by oxygen accepting electrons from FADH_2_, producing H_2_O_2_ and FAD. In the presence of PB, H_2_O_2_ is reduced, and PB in its oxidized form (PB_ox_) is generated. Subsequently, PB_ox_ is reduced to PB_red_ at the electrode (Figure 6f and Figure S3). CV curves of the resultant electrode in PBS containing different concentrations of glucose were recorded (Figure 6c). As can be seen, CV curves in a glucose-containing solution show a higher reduction current and lower oxidation current, demonstrating that the GOx on the working electrode catalyzes glucose.

Glucose + GOx(FAD) → Gluconic acid+ GOx(FADH_2_) (1)

GOx(FADH_2_) + PB_ox_ → GOx(FAD) + PB_red_ (2)

GOx(FADH_2_) + O_2_ → GOx(FAD) + H_2_O_2_ (3)

PB_red_ + H_2_O_2_ → PB_ox_ + H_2_O + O_2_ (4)

PB_red_ ↔ PB_ox_ + e^−^ (5)

To further evaluate the performance of the constructed biosensor, chronoamperometry was employed to measure the analytical performance (Figure 6d). Figure 6e shows the linear relationship between the current response and the logarithmic value of glucose concentration, and the electrode exhibits a distinguishable response in glucose concentration. Good linearity with an R^2^ of 0.9886 and sensitivity of 31.94 μA (lg(mM))^−1^ cm^−2^ were observed at concentrations ranging from 0 to 30 mM, and the detection limit (LOD) calculated at S/N = 3 was 1 mM. This range includes the average glucose concentration of human ISF and diabetes patients [18]. Meanwhile, the current response of the electrode to glucose and other interfering substances (UA, AA) was measured at the same voltage (0.05 V), as shown in Figure 6f. It can be observed that this electrode exhibits high selectivity towards glucose due to the presence of GOx and PB, and its response to interfering substances can be ignored. We conducted continuous tests on the electrode’s current response to glucose over 30 days to evaluate the sensor’s long-term performance. Minimal current changes were obtained post-30 days, demonstrating the sensor’s good long-term stability (Figure S4). When bending is applied to the whole sensor, it can still conduct signals well (Figure S6). It can detect glucose under different bending conditions. To compare our results with others, we have listed a few publications with electrodes for glucose sensing in Table 1. In summary, the as-prepared all-fiber glucose sensor can be well applied in further wearable and implantable monitoring.

## 4. Conclusions

In this work, we designed and fabricated an all-fiber electrochemical electrode based on electrospinning. A Au conductive network was formed on PU fiber by thermal evaporation. Subsequently, electrochemical deposition was employed to form AuNFs and wrinkled PB on the electrode surface to improve the electrode’s electrochemical performance. Then, GOx-AuNPs-chitosan was immobilized on the electrode to detect glucose. A linear response was obtained within the concentration range of 1–30 mM, with a sensitivity of 31.94 μA (lg(mM))^−1^ cm^−2^ and LOD of 1 mM (S/N = 3). The sensor also exhibited good anti-interference performance against UA and AA. Therefore, the proposed flexible sensor is a reliable device for health monitoring, providing new insights for fiber-based electrochemical sensors. Modification with different functional materials can achieve monitoring of different substances, and it is expected to achieve multimodal sensing.

## Figures and Tables

**Table 1:** Comparison of different glucose electrodes.

Reference	Electrode and Materials	Sensitivity	LOD	Concentration Range
This work	Electrospun electrode w/AuNFs/PB	31.94 μA (lg(mM))^−1^ cm^−2^	1.01 mM	1–30 mM
[19]	Solid MN w/Au/pTCA/GOx	0.22 μA (mM)^−1^ cm^−2^	19.4 μM	0.05–20 mM
[20]	Electrospun electrode w/PEDOT/GOx	74.22 μA (mM)^−1^ cm^−2^	2.9 μM	0–4.5 mM
[21]	Graphene/CNT fiber w/GOx	14.45 μA (mM)^−1^ cm^−2^	0.06 μM	0–30 mM
[22]	Electrospun fiber w/PEDOS: PSS	0.386 μA (mM)^−1^ cm^−2^	3.31 mM	0–30 mM
[23]	MN array w/Au/Fc-PAMAM/GOx	0.1622 μA (mM)^−1^ cm^−2^	660 μM	1–9 mM
[24]	Screen printing electrode w/PPG/GOx/PU	12.69 μA (mM)^−1^ cm^−2^	No mention	1–30 mM

Notes: MN, microneedle; pTCA, polyterthiophene carboxylic acid; PEDOT, poly(3,4-ethylenedioxythiophene); CNT, carbon nanotube; Fc-PAMAM, ferrocene-cored poly(amidoamine); PPG, poly (N-phenylglycine).

## Data Availability

Data will be made available upon request.

## Supplementary Materials

The following supporting information can be downloaded at https://www.mdpi.com/article/10.3390/s24144580/s1, Figure S1: The fiber widths of the electrodes after electrodeposition at different HAuCl_4_ concentrations. Figure S2: The fiber widths of the electrodes after electrodeposition at different HAuCl_4_ concentrations. Figure S3: Changes in sensor current response under different test conditions. Figure S4: The electrode’s current response curve to glucose in the 30-day range. Figure S5: Mechanical stability of electrodes. Figure S6: CV curves of the electrode under different bending conditions in the solution with 30 mM glucose.

## References

[1] Min J., Tu J., Xu C., Lukas H., Shin S., Yang Y., Solomon S.A., Mukasa D., Gao W. (2023). Skin-Interfaced Wearable Sweat Sensors for Precision Medicine. Chem. Rev..

[2] Luo Y., Abidian M.R., Ahn J.-H., Akinwande D., Andrews A.M., Antonietti M., Bao Z., Berggren M., Berkey C.A., Bettinger C.J. (2023). Technology Roadmap for Flexible Sensors. ACS Nano.

[3] Song Y., Tay R.Y., Li J., Xu C., Min J., Shirzaei Sani E., Kim G., Heng W., Kim I., Gao W. (2023). 3D-Printed Epifluidic Electronic Skin for Machine Learning–Powered Multimodal Health Surveillance. Sci. Adv..

[4] Ju J., Hsieh C.-M., Tian Y., Kang J., Chia R., Chang H., Bai Y., Xu C., Wang X., Liu Q. (2020). Surface Enhanced Raman Spectroscopy Based Biosensor with a Microneedle Array for Minimally Invasive In Vivo Glucose Measurements. ACS Sens..

[5] Li Q.-F., Chen X., Wang H., Liu M., Peng H.-L. (2023). Pt/MXene-Based Flexible Wearable Non-Enzymatic Electrochemical Sensor for Continuous Glucose Detection in Sweat. ACS Appl. Mater. Interfaces.

[6] Yin L., Cao M., Kim K.N., Lin M., Moon J.-M., Sempionatto J.R., Yu J., Liu R., Wicker C., Trifonov A. (2022). A Stretchable Epidermal Sweat Sensing Platform with an Integrated Printed Battery and Electrochromic Display. Nat. Electron..

[7] Bai J., Liu D., Tian X., Wang Y., Cui B., Yang Y., Dai S., Lin W., Zhu J., Wang J. (2024). Coin-Sized, Fully Integrated, and Minimally Invasive Continuous Glucose Monitoring System Based on Organic Electrochemical Transistors. Sci. Adv..

[8] Niu J., Lin S., Chen D., Wang Z., Cao C., Gao A., Cui S., Liu Y., Hong Y., Zhi X. (2023). A Fully Elastic Wearable Electrochemical Sweat Detection System of Tree-Bionic Microfluidic Structure for Real-Time Monitoring. Small.

[9] Chen J., Tao X., Xu X., Sun L., Huang R., Nilghaz A., Tian J. (2024). Making Commercial Bracelet Smarter with a Biochemical Button Module. Biosens. Bioelectron..

[10] Gao W., Emaminejad S., Nyein H.Y.Y., Challa S., Chen K., Peck A., Fahad H.M., Ota H., Shiraki H., Kiriya D. (2016). Fully Integrated Wearable Sensor Arrays for Multiplexed in Situ Perspiration Analysis. Nature.

[11] Tehrani F., Teymourian H., Wuerstle B., Kavner J., Patel R., Furmidge A., Aghavali R., Hosseini-Toudeshki H., Brown C., Zhang F. (2022). An Integrated Wearable Microneedle Array for the Continuous Monitoring of Multiple Biomarkers in Interstitial Fluid. Nat. Biomed. Eng..

[12] Saha T., Del Caño R., Mahato K., De la Paz E., Chen C., Ding S., Yin L., Wang J. (2023). Wearable Electrochemical Glucose Sensors in Diabetes Management: A Comprehensive Review. Chem. Rev..

[13] Krouwer J.S. (2023). Adverse Event Causes from 2022 for Four Continuous Glucose Monitors. J. Diabetes Sci. Technol..

[14] Wang X.-X., Yu G.-F., Zhang J., Yu M., Ramakrishna S., Long Y.-Z. (2021). Conductive Polymer Ultrafine Fibers via Electrospinning: Preparation, Physical Properties and Applications. Prog. Mater. Sci..

[15] Yan L., Miao K., Ma P., Ma X., Bi R., Chen F. (2021). A Feasible Electrochemical Biosensor for Determination of Glucose Based on Prussian Blue—Enzyme Aggregates Cascade Catalytic System. Bioelectrochemistry.

[16] Reneker D.H., Yarin A.L. (2008). Electrospinning Jets and Polymer Nanofibers. Polymer.

[17] Siciliano G., Alsadig A., Chiriacò M.S., Turco A., Foscarini A., Ferrara F., Gigli G., Primiceri E. (2024). Beyond Traditional Biosensors: Recent Advances in Gold Nanoparticles Modified Electrodes for Biosensing Applications. Talanta.

[18] Heikenfeld J., Jajack A., Feldman B., Granger S.W., Gaitonde S., Begtrup G., Katchman B.A. (2019). Accessing Analytes in Biofluids for Peripheral Biochemical Monitoring. Nat. Biotechnol..

[19] Kim K.B., Lee W.-C., Cho C.-H., Park D.-S., Cho S.J., Shim Y.-B. (2019). Continuous Glucose Monitoring Using a Microneedle Array Sensor Coupled with a Wireless Signal Transmitter. Sens. Actuators B Chem..

[20] Çetin M.Z., Camurlu P. (2018). An Amperometric Glucose Biosensor Based on PEDOT Nanofibers. RSC Adv..

[21] Yao Y., Chen J., Guo Y., Lv T., Chen Z., Li N., Cao S., Chen B., Chen T. (2021). Integration of Interstitial Fluid Extraction and Glucose Detection in One Device for Wearable Non-Invasive Blood Glucose Sensors. Biosens. Bioelectron..

[22] Seufert B., Thomas S., Takshi A. (2024). Stretchable Nanofiber-Based Felt as a String Electrode for Potential Use in Wearable Glucose Biosensors. Sensors.

[23] Dervisevic M., Alba M., Yan L., Senel M., Gengenbach T.R., Prieto-Simon B., Voelcker N.H. (2022). Transdermal Electrochemical Monitoring of Glucose via High-Density Silicon Microneedle Array Patch. Adv. Funct. Mater..

[24] Jin X., Li G., Xu T., Su L., Yan D., Zhang X. (2022). Fully Integrated Flexible Biosensor for Wearable Continuous Glucose Monitoring. Biosens. Bioelectron..

