# Supplementary material for: All-Fiber Flexible Electrochemical Sensor for Wearable Glucose Monitoring
Source: Sensors (Basel). 2024 Jul 15;24(14):4580. doi: 10.3390/s24144580 (PMC11281184; doi:10.3390/s24144580)
Supplement: Supplementary file 1 [file sensors-24-04580-s001.zip › sensors-3046571-supplementary.pdf]

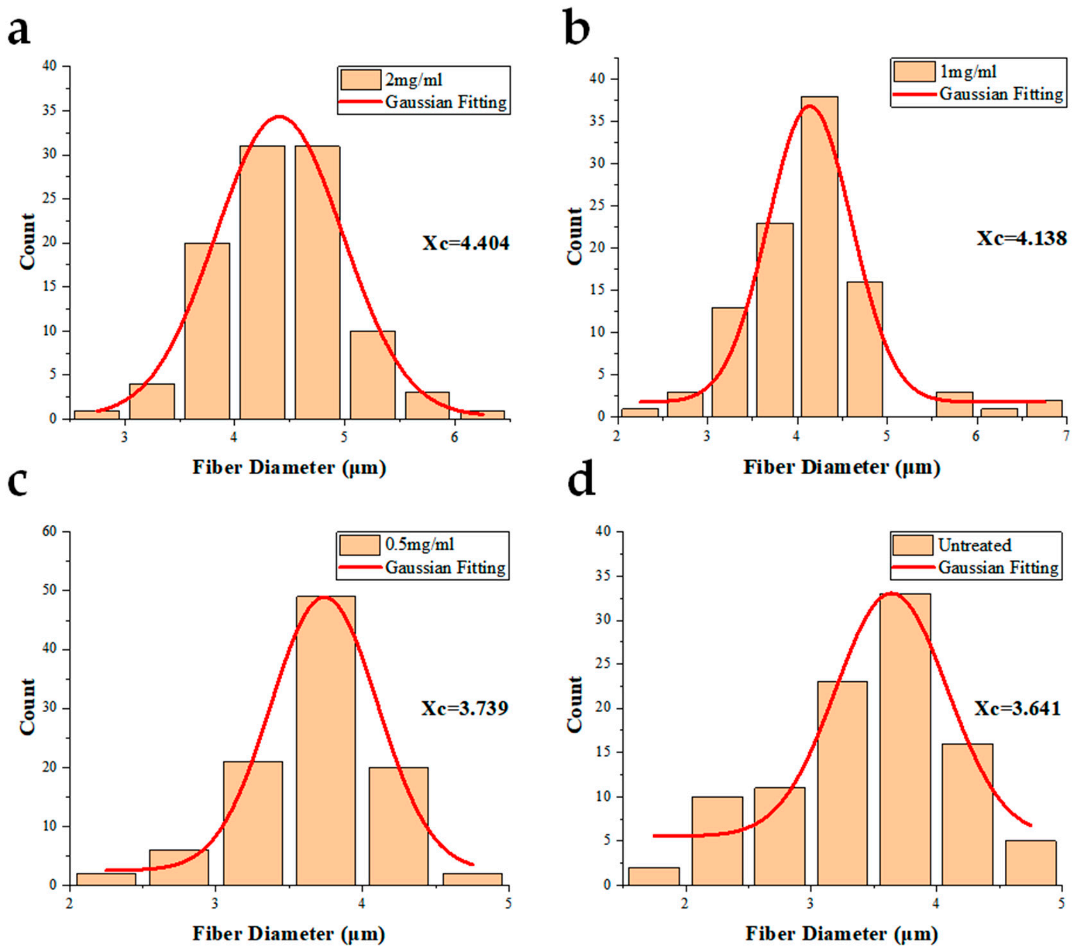

Figure S1. The fiber widths of the electrodes after electrodeposition at different $HAuCl_4$ concentrations: (a) 2mg/ml; (b) 1mg/ml; (c) 0.5mg/ml; (d) untreated.

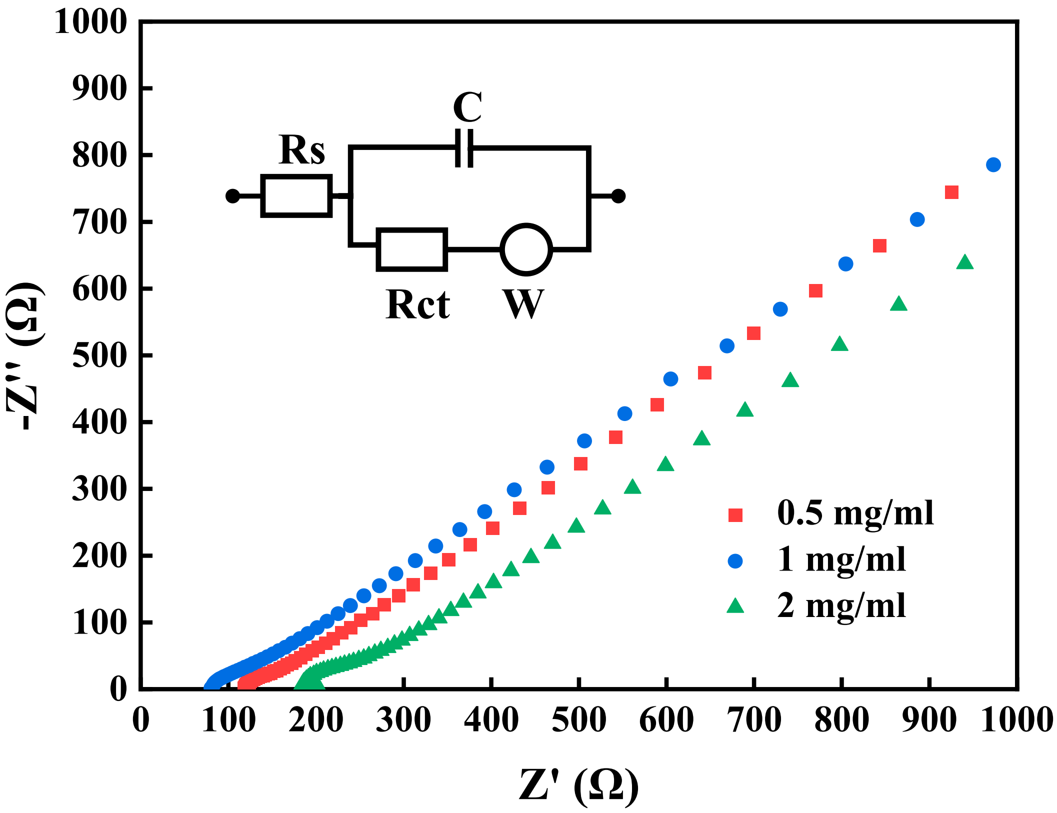

Figure S2. The fiber widths of the electrodes after electrodeposition at different $HAuCl_4$ concentrations: (a) 2mg/ml; (b) 1mg/ml; (c) 0.5mg/ml; (d) untreated.

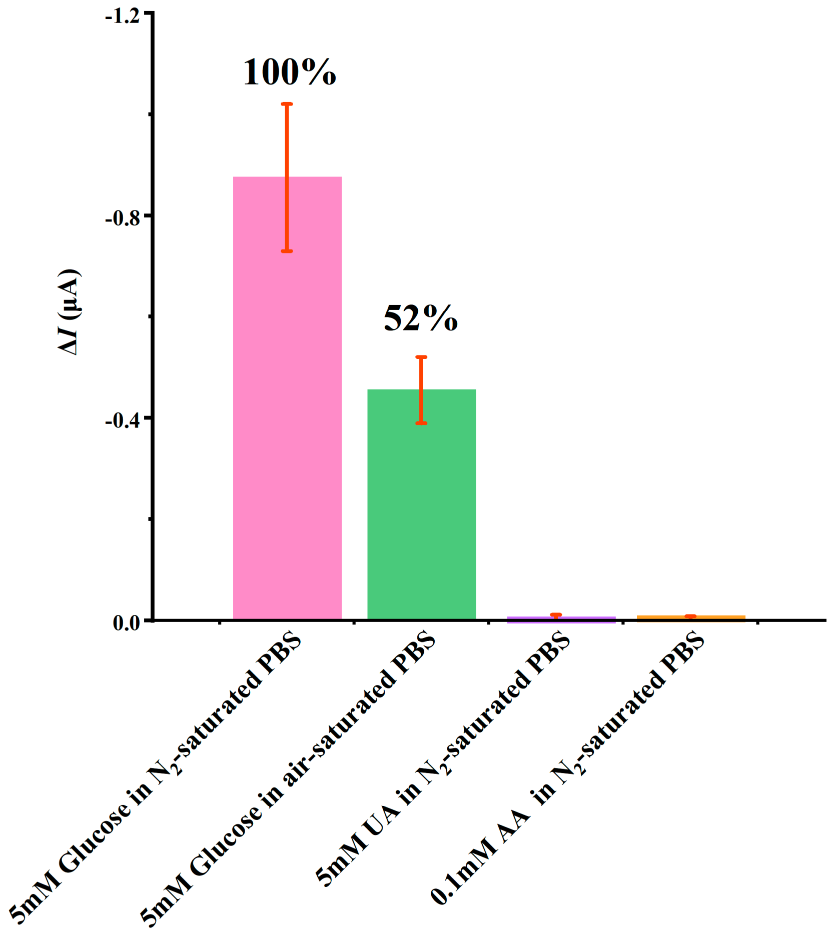

Figure S3. Changes in sensor current response under different test conditions.

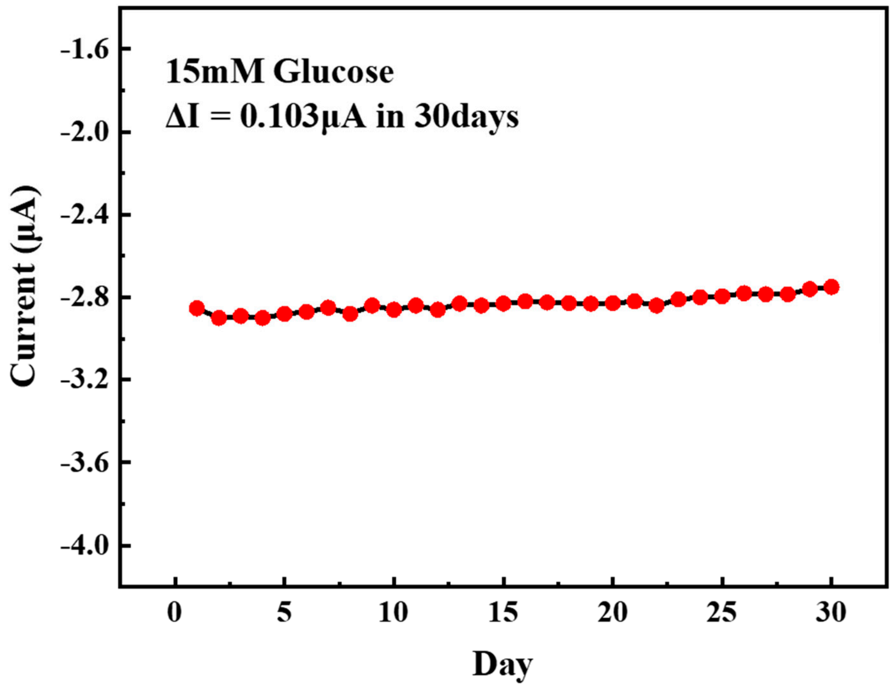

Figure S4. The electrode's current response curve to glucose in the 30-day range.

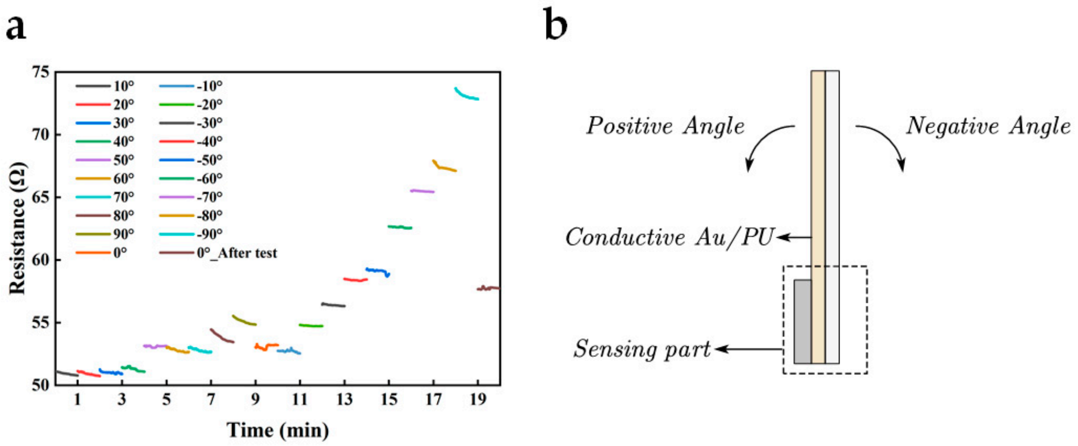

Figure S5. Mechanical stability of electrodes. (a) Change in resistance of electrodes under different bending conditions; (b) d~~D~~iagram of test configuration. The sensing part is carefully affixed to a polyethylene terephthalate (PET) substrate, and~~while~~ the conductive path is similarly affixed to another PET substrate. In this arrangement, the angle delineated by the two substrates, when fully aligned within the same plane, is defined as 0°.

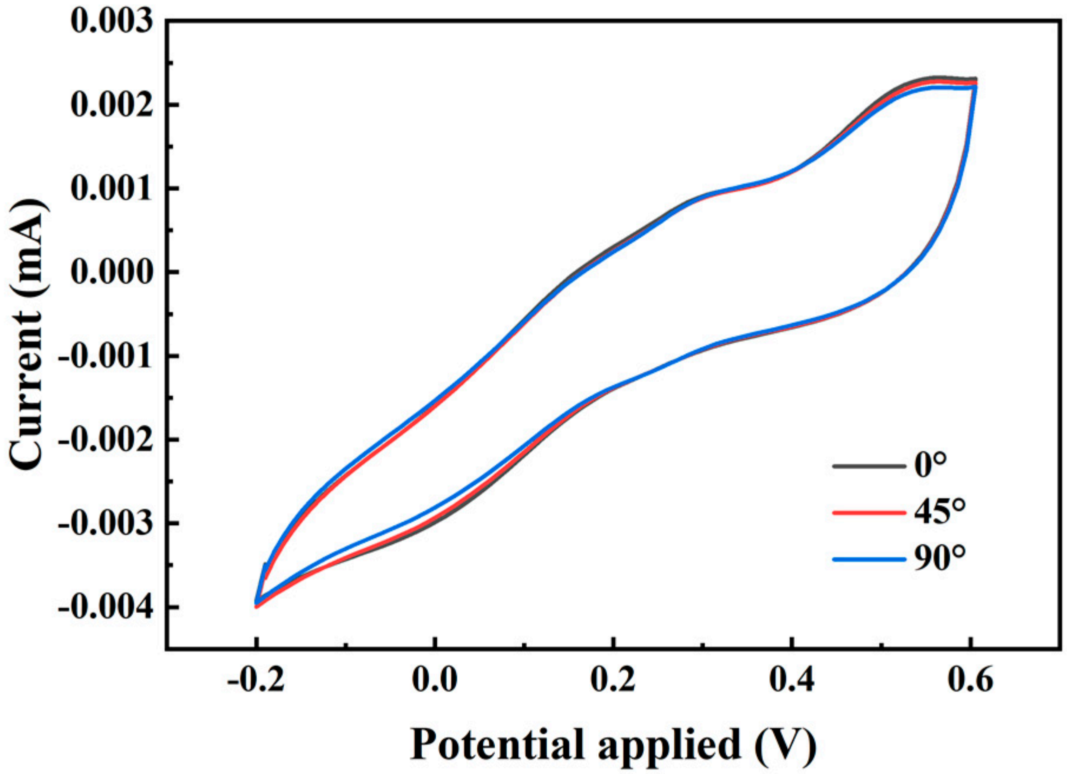

Figure S6. CV curves of the electrode under different bending conditions (45° and 90°) in the solution with 30 mM glucose.
